# Sarcoidosis-Related Uveitis: A Review

**DOI:** 10.3390/jcm12093194

**Published:** 2023-04-29

**Authors:** Stéphane Giorgiutti, Robin Jacquot, Thomas El Jammal, Arthur Bert, Yvan Jamilloux, Laurent Kodjikian, Pascal Sève

**Affiliations:** 1 Department of Clinical Immunology and Internal Medicine, National Center for Systemic Autoimmune Diseases (CNR RESO), Strasbourg University Hospital, 67000 Strasbourg, France; 2 INSERM UMR-S1109, Université de Strasbourg, 67000 Strasbourg, France; 3 Department of Internal Medicine, Croix-Rousse University Hospital, Hospices Civils de Lyon, 69004 Lyon, France; 4 Faculté de Médecine et de Maïeutique Lyon-Sud—Charles Mérieux, Université de Lyon, 69000 Lyon, France; 5 Laboratory of Tissue Biology and Therapeutic Engineering, CNRS UMR5305, IBCP, University of Lyon, 69007 Lyon, France; 6 Department of Ophthalmology, Croix-Rousse University Hospital, Hospices Civils de Lyon, 69004 Lyon, France; 7 UMR5510 MATEIS, CNRS, INSA Lyon, Université de Lyon 1, 69100 Villeurbanne, France; 8 Pôle IMER, Hospices Civils de Lyon, 69002 Lyon, France; 9 The Health Services and Performance Research (EA 7425 HESPER), Université de Lyon, 69003 Lyon, France

**Keywords:** sarcoidosis, uveitis, ocular sarcoidosis, granuloma

## Abstract

Sarcoidosis is an inflammatory disease that involves the eyes in 10–55% of cases, sometimes without systemic involvement. All eye structures can be affected, but uveitis is the most common ocular manifestation and causes vision loss. The typical ophthalmological appearance of these uveitis is granulomatous (in cases with anterior involvement), which are usually bilateral and with synechiae. Posterior involvement includes vitritis, vasculitis and choroidal lesions. Tuberculosis is a classic differential diagnosis to be wary of, especially in people who have spent time in endemic areas. The diagnosis is based on histology with the presence of non-caseating epithelioid granulomas. However, due to the technical difficulty and yield of biopsies, the diagnosis of ocular sarcoidosis is often based on clinico-radiological features. The international criteria for the diagnosis of ocular sarcoidosis have recently been revised. Corticosteroids remain the first-line treatment for sarcoidosis, but up to 30% of patients require high doses, justifying the use of corticosteroid-sparing treatments. In these cases, immunosuppressive treatments such as methotrexate may be introduced. More recent biotherapies such as anti-TNF are also very effective (as they are in other non-infectious uveitis etiologies).

## 1. Introduction

Sarcoidosis is a complex and heterogeneous granulomatous systemic inflammatory disease. The characteristic histological lesion is the presence of non-caseating epithelioid giant cell granulomas in the tissues. The first clinical description dates from the second half of the 19th century and is due to Sir Jonathan Hutchinson [1]. However, to date, the pathophysiology of the disease remains poorly understood. It could be at the crossroads of inflammatory processes induced by environmental factors, particularly infectious ones such as *Cutibacterium acnes*, on a predisposing genetic terrain [2,3]. The ACCESS study revealed a familial risk of sarcoidosis with an estimated odds ratio of 5.8 (confidence interval confidence interval [CI]: 2.1–15.9) for siblings and 3.8 (95% CI: 1.2–11.3) for first degree relatives [4]. More recently, in two studies from Northern Europe, the heritability of sarcoidosis has been estimated at between 39 and 66% [5,6]. The typical involvement of the disease is thoracic with the presence of mediastinal lymph nodes +/− parenchymal lung involvement. Ten to fifty-five percent of patients develop ophthalmological damage, making the eye the primary extra-thoracic organ affected [7,8,9,10,11]. All ocular tissues can be affected, including the lacrymal glands and the optic nerve [12,13,14,15,16]. Sarcoid uveitis, which is the subject of this review, is the most common ocular condition in sarcoidosis and can be sight-threatening, particularly in the case of posterior involvement [17]. It affects up to 20–30% of sarcoidosis patients [18]. This review will focus on the most recent data, including the classification criteria for sarcoidosis-associated uveitis from the Standardization of Uveitis Nomenclature (SUN) working group and the recommendations for the management of ocular sarcoidosis from the International Workshop on Ocular Sarcoidosis (IWOS).

## 2. Epidemiology

Globally, sarcoidosis incidence ranges from 0.48 to 11.4 cases per 100,000 people per year [19]. African Americans have the higher incidence of sarcoidosis, reaching 17.8 per 100,000 per year [20,21]. The prevalence of ocular sarcoidosis varies from 10–50% in Caucasian studies [22,23,24,25,26]. Ocular sarcoidosis is more prevalent in Asian population. In Japan, sarcoidosis has become the leading cause of uveitis, accounting for approximately 15% of all cases [10,27]. Data regarding ocular sarcoidosis in Africans Americans are scarce; however, compared with Caucasians, these patients seem to be younger at ophthalmological presentation with uveitis and/or adnexal granuloma [28]. Uveitis remains the most frequent ocular condition apart from sicca syndrome [29]. In a population of patients with uveitis, the prevalence of sarcoidosis depends on demographic factors (age, gender, ethnicity), the diagnostic investigations employed (i.e., positron emission tomography) and the type of recruitment (tertiary care center or not) [28,28,29,30,31]. Sarcoidosis accounts for 2 to 17% of cases of uveitis referred to a tertiary center [32,33,34,35,36]. Uveitis is the presenting feature of sarcoidosis in 60–80% of cases [37,38,39]. Sarcoidosis uveitis as a manifestation of the disease remains a strictly ocular disease in more than three-quarters of cases [38,40].

## 3. Phenotypes of Patients with Sarcoid Uveitis

Two phenotypes are classically described: The first one concerns young subjects from 20 to 30 years old of varied ethnic origin, with more often acute uveitis associated with extra-ophthalmological manifestations. The second involves mostly women over 50 years of age of European origin, with more frequently isolated chronic uveitis [41,42]. We have recently identified a third cluster of patients corresponding to patients of European origin that is older than the first group; here, the proportion of acute and chronic uveitis is equivalent and the visual prognosis is better than in the classic cluster of young patients [43]. However, these studies are conducted in European countries and should be interpreted with caution for other populations.

Using cluster analysis, Schupp et al. showed an association of ocular, cardiac, skin and central nervous system manifestations [7]. In accordance, Van Swol et al. recently reported that 16% of the patients with ocular sarcoidosis had signs of cardiac sarcoidosis on electrocardiogram at the time of their ocular sarcoidosis diagnosis [44]. In contrast, in our center, we showed that out of 294 patients with sarcoid uveitis only 2.4% of them developed cardiac involvement [45]. Niederer et al. also reported 4.4% of cardiac sarcoid in their retrospective cohort of sarcoid uveitis [39]. However, special attention should be paid to patients with previously diagnosed sarcoidosis or those who develop systemic sarcoidosis during follow-up.

## 4. Clinical Manifestations of Sarcoid Uveitis

Uveitis is defined as inflammation of the uveal tract. In sarcoidosis, uveitis can be of any anatomical type: anterior, intermediate, posterior or panuveitis [46]. Anterior uveitis is by far the most common, accounting for 41–81% of sarcoid uveitis [8,47]. Of note, in tertiary centers, which manage the most severe forms, panuveitis is the most frequent presentation in studies [37,38,48]. It is typically bilateral and granulomatous with a symmetrical course in both eyes; however, it remains unilateral in up to 25% of cases [29,49]. The SUN working group published classification criteria for sarcoid uveitis in 2021 [50]. The IWOS group also proposed seven ophthalmological signs suggestive of sarcoid uveitis and specific classification criteria [51]. The 2020 American Thoracic Society recommendations suggest a systematic ophthalmological examination of any patient with sarcoidosis, even in the absence of ophthalmological symptoms [52]. However, the level of evidence remains low, and a recent prospective study of 49 patients in the US did not identify a benefit for the screening of asymptomatic patients [53].

### 4.1. Anterior Uveitis

**Figure 1:**
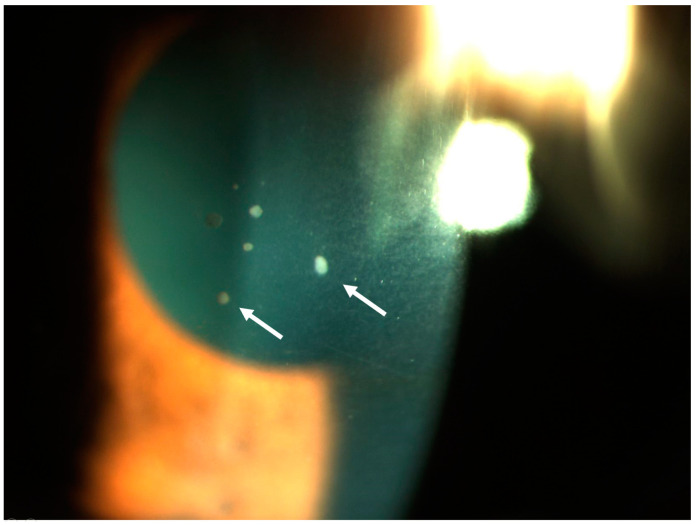
Acute granulomatous anterior uveitis with active retrodescemetic precipitates (white arrows).

Anterior uveitis can be acute (with an abrupt onset and duration of less than three months) but is more often chronic (prolonged with relapses less than three months after cessation of treatment) [54]. The inflammation takes the form of an iritis, iridocyclitis or anterior hyalitis [54]. Uveitis may be associated with increased intraocular pressure, either due to the ocular inflammation itself or caused by the treatment [50,55]. Typically, there are anterior (between the cornea and the iris) and posterior synechiae (between the iris and the lens) [50]. Sarcoid uveitis is most often granulomatous, which is characterized by large mutton-fat keratic precipitates or iridal nodules located either at the pupil margin (Koeppe nodules) or in the iridal stroma (Busacca nodules) (Figure 1). However, granulomatous uveitis is not pathognomonic of sarcoidosis, as other etiologies such as tuberculosis can also be characterized by granulomatous uveitis. Furthermore, in some studies, more than half of the patients have non-granulomatous uveitis, especially in cases of Löfgren syndrome [28,50,56].

### 4.2. Intermediate Uveitis

In intermediate uveitis, inflammation occurs mainly in the vitreous humor as pars planitis, posterior cyclitis or hyalitis [54,57]. Intermediate uveitis is most often idiopathic, but sarcoidosis accounts for 7–18% of this anatomical type of uveitis, making it a common etiology along with multiple sclerosis [35,58,59]. The most common features of intermediate uveitis in sarcoidosis are vitreous “snowballs” that may be organized into a “string of pearls”. The leading cause of vision loss in patients with intermediate uveitis is cystoid macular edema, followed by vitreous opacity, epiretinal membrane, optic neuritis and glaucoma [58].

### 4.3. Posterior Uveitis

**Figure 2:**
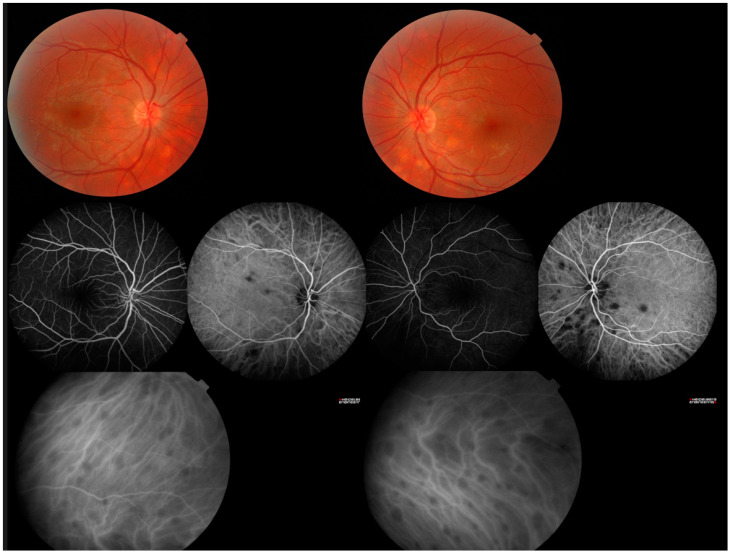
Central and peripheral bilateral multifocal choroiditis.

Posterior uveitis concern inflammation involving the retina and/or choroid [46]. Fundus examination is a key part of the clinical examination, but the use of additional examinations such as optical coherence tomography (OCT) and angiography are very useful. Posterior uveitis accounts for 5–28% of ocular sarcoidosis [28,29,47,49,60]. Although less common than anterior involvement, it is more threatening to the patient’s vision [61]. Authentic choroidal granulomas in peripheral retina or around the optic nerve have been described but multifocal choroiditis (Figure 2) is much more common [50,62,63]. Both lesions can evolve into atrophic scars of the pigmentary epithelium. Retinal and pre-retinal nodules have rarely been reported as the sole posterior manifestations of ocular sarcoidosis without choroidal involvement [64]. In severe forms, these granulomas can lead to exudative retinal detachment [65]. OCT/angiography may be useful to visualize changes in granuloma formation and to assess microvascular and perfusion impairments [66,67]. Indeed, retinal vasculitis is often associated with sarcoidosis. Ten to seventeen percent of patients suffer from periphlebitis [50]. The classic perivascular sheathing and infiltrates, called “candle wax dripping”, is rare and can sometimes only be identified by fluorescein angiography [12]. This condition is classically seen in the acute phase of uveitis and is usually associated with a poorer visual prognosis and more frequent relapses [68]. Some vasculitis are associated with vascular occlusions (especially venous) that may be complicated by retinal neovascularization in up to 5% of cases (in association with ischemia and chronic inflammation) [69]. Arterial involvement in sarcoidosis is scarce [70].

### 4.4. Panuveitis

Panuveitis affects all structures of the eye and combines all the lesions we have already described [46]. They are the most common form of uveitis in tertiary centers and are estimated to represent 37% of sarcoid uveitis by the SUN working group [29,50]. Sarcoidosis is the most frequently systemic disease associated with panuveitis, ahead of tuberculosis and Behçet’s disease [35,71,72].

### 4.5. Ocular Complications

**Figure 3:**
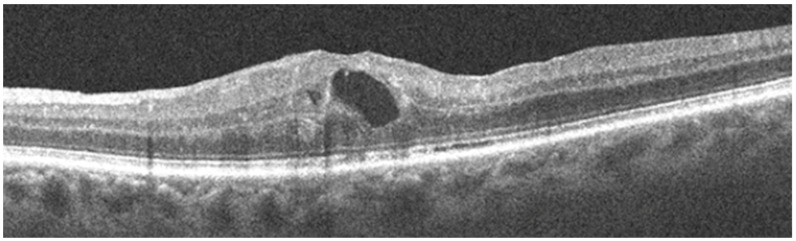
Cystoid macular edema in spectral domain by optical coherence tomography.

Even anterior uveitis can lead to ocular complications, including band keratopathy, cataract and glaucoma. These complications are both secondary to the inflammatory process and also iatrogenic under corticosteroids [41]. More seriously, cystoid macular edema (Figure 3) is the main cause of vision loss in sarcoid uveitis [37]. Epiretinal membranes may occur in cases of severe vitreoretinal inflammation and may be responsible for retinal traction, resulting in retinal tears and rhegmatogenous retinal detachment [73].

## 5. Diagnostic Approach

### 5.1. Sarcoidosis: A Challenging Diagnosis

**Figure 4:**
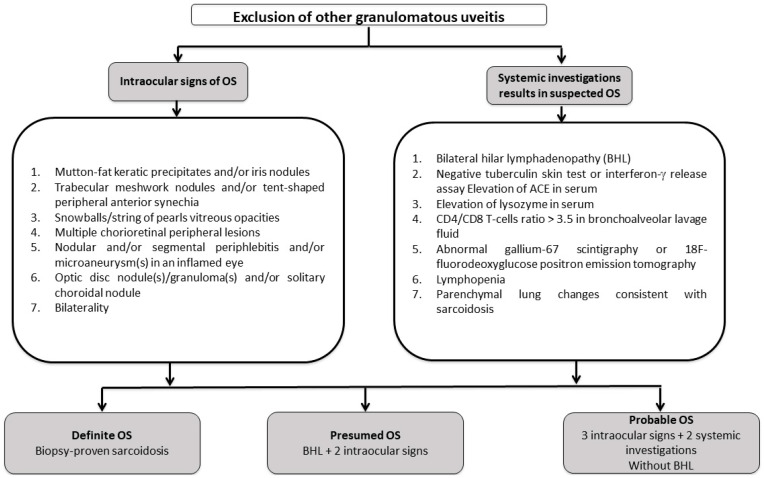
Revised diagnostic criteria for ocular sarcoidosis (OS) as recommended by the “International Workshop on Ocular Sarcoidosis (IWOS)”, adapted from [51]. Abbreviations: BHL: bilateral hilar lymphadenopathy; ACE: angiotensin converting enzyme; OS: ocular sarcoidosis.

Again, no clinical feature of the uveitis is specific to sarcoidosis. From a strictly ophthalmological point of view, a diagnosis of uveitis must always lead to the elimination of a masquerade syndrome with particularly serious consequences, especially in the case of lymphoma [74,75]. Furthermore, granulomatous uveitis are also associated with tuberculosis, syphilis, multiple sclerosis, Vogt–Koyanagi–Harada syndrome, toxoplasmosis and herpetic uveitis [76]. Choroidal granulomas are often seen in tuberculosis, making this infectious disease a challenging differential diagnosis [77,78]. No “simple” marker is available to make the diagnosis of sarcoidosis and therefore systemic investigations are required. The gold standard for the diagnosis of sarcoidosis is histological evidence of non-caseating epithelioid giant-cell granulomas [79]. However, intraocular tissue biopsy is associated with the risk of potentially sight-threatening lesions. Furthermore, the diagnostic value of blind conjunctival biopsies remains controversial [80,81,82]. In practice, the diagnosis of sarcoid uveitis is therefore frequently based on a combination of clinical and paraclinical data. International criteria for the diagnosis of ocular sarcoidosis were proposed in 2009 as a result of the first IWOS [83]. Revised criteria were then proposed in 2017 (Figure 4), as the original criteria had low sensitivity, with the exception of bilateral hilar adenopathy [51]. In addition to definite sarcoidosis still requiring histological evidence, the group defined presumed ocular sarcoidosis in the presence of bilateral hilar adenopathies and probable ocular sarcoidosis in the absence of these adenopathies [51]. More recently, a SUN working group has proposed diagnostic criteria for 25 uveitis entities including sarcoidosis uveitis (Table 1) that combines a compatible ophthalmological presentation with evidence of sarcoidosis (in the form of histological evidence or adenopathy) [50].

In all cases of uveitis, a minimal work-up should be performed with a blood count, C-reactive protein, syphilis serology and tuberculin skin tests (or IFN-γ release assays, IGRA) and chest imaging [84]. In addition, a positive tuberculin test or IGRA is an exclusion criteria in the SUN classification [50]. Nevertheless, 12% of patients with sarcoid uveitis meeting the inclusion criteria had a positive IGRA in France, a low-endemic country for tuberculosis [85]. Empirical anti-tuberculosis therapy should be started in doubtful cases [86].

### 5.2. In Search of Serum Predictive Biomarkers for Sarcoidosis

To date, no biomarker is robust enough to make a diagnosis of sarcoidosis (Table 2). Anti-retinal antibodies have been described in ocular sarcoidosis as in other types of uveitis; however, their sensitivity and specificity currently remain insufficient to recommend them [87]. A simple biological finding, lymphopenia, appears in the 2017 IWOS criteria [51]. In the population with a first episode of uveitis, Groen-Hakan et al. showed the sensitivity and specificity of lymphopenia to be 75% and 77%, respectively, taking a cut-off of 1.5 × 10^9^/L [88]. Angiotensin-converting enzyme (ACE) is probably the best known and most used marker described in the 1970s [89]. The sensitivity varies among the series from 38.2–84% and the specificity from 83–97.8% for ACE [84,90,91]. The large variability in the sensitivity of ACE is probably due to different thresholds for positivity in different studies. In a retrospective study of 709 patients with undifferentiated uveitis, 43 subjects (6.1%) had high serum ACE. Of these, 29 (67.4%) had systemic sarcoidosis [91]. In addition, patients treated with ACE inhibitors have uninterpretable results [92]. The combination of elevated serum ACE and lymphopenia more convincingly suggests sarcoid uveitis than these investigational tests alone, especially in patients with granulomatous uveitis (positive predictive value of 73.3%), whereas the absence of these markers corresponds to a high negative predictive value (negative predictive value of 89.5%) [93]. The serum lysozyme assay has an estimated sensitivity of 60–78% and a specificity of 76–95% [84]. However, lysozyme may be increased in patients with latent tuberculosis and latent syphilis, and its interpretation alone should be treated with caution [94]. The other studied markers of sarcoidosis are not available in clinical routine. Nevertheless, we can mention the soluble interleukin-2 receptor (sIL-2R) [95]. In patients with ocular sarcoidosis, this marker would have better sensitivity (69.2 to 94%) and specificity (64 to 98%) than ACE [95,96,97,98]. sIL-2R may correlate with disease activity and predict relapse after treatment discontinuation [99]. Other biomarkers are being investigated in sarcoidosis such as chitotriosidase and Krebs von den Lungen (KL-6); however, in the absence of clinical implications, they will not be detailed here [100,101,102]. All of these serum biomarkers alone are insufficient to make a diagnosis of sarcoidosis, and it is their combination with morphological examinations that increases the diagnostic efficiency [42,103].

### 5.3. Imaging Modalities

Chest CT is probably the most frequently used imaging test in sarcoidosis screening at present, supplanting chest X-rays [105]. Parenchymal lung abnormalities consistent with sarcoidosis are now included in the IWOS criteria for ocular sarcoidosis, provided that imaging is reviewed by specialized pulmonologists or radiologists [51]. More recently, nuclear imaging with 18F-fluorodeoxyglucose positron emission tomography (18F-FDG PET) has become an imaging modality of choice in the diagnosis and management of sarcoidosis, although large-scale prospective studies will be needed to clarify its place in the diagnostic work-up [106]. 18F-FDG PET would be of interest: (1) in cases of suspected extra-pulmonary involvement, such as neurosarcoidosis or cardiac sarcoidosis, where it can help to define a target for biopsy, (2) in cases of pulmonary fibrosis to assess active lesions that may regress with anti-inflammatory treatment, (3) in cardiac sarcoidosis to assess the response to treatment (cardiac 18F-FDG-PET), (4) in the most complex cases to assess the therapeutic response and the relapse risk [107]. Older age at diagnosis, the presence of posterior synechiae and increased ACE levels are significantly associated with an abnormal 18F-FDG PET [108,109]. In this study, although chest CT was normal, 30% of patients with suspected sarcoid uveitis had hypermetabolic foci on 18F-FDG PET [109]. We must, however, remain cautious about the contribution of 18F-FDG PET in comparison with chest CT, particularly in the differential diagnosis with ocular tuberculosis, where conflicting data exist in tuberculosis endemic areas [110].

### 5.4. Invasive Investigations

Several studies have reported the value of bronchoalveolar lavage (BAL) for the diagnosis of sarcoid uveitis [25]. The sensitivity of BAL is estimated at 63% in patients with histologically proven sarcoidosis, whereas the specificity is 75% [94]. Lymphocytic alveolitis (>15%) with a predominance of CD4 T cells (ratio CD4/CD8 > 3.5) can be demonstrated even in the absence of radiological abnormalities [25,60,111,112]. However, the biopsy is never positive in cases with a normal CT scan [111]. Other studies have reported that CD4/CD8 ratios in other biological fluids (e.g., vitreous fluid) were significantly higher in patients with sarcoidosis compared with other causes of uveitis. [8,113,114]. Work such as that by De Simone et al. is attempting to identify cytokine profiles in the aqueous humor to guide the diagnosis between sarcoidosis and tuberculosis [115]. Proteomic analysis of vitreous humor samples in ocular sarcoidosis are also performed in search of new biomarkers [116]. However, the highly invasive nature of this type of sample will limit its widespread use. Minor salivary gland biopsy (MSGB) is not mentioned in the revised IWOS criteria. The diagnostic performance of which is low in ocular sarcoidosis with a sensitivity of 5.2 and 3%, respectively, in studies [117,118]. Furthermore, MSGB does not exclude tuberculosis, which is an integral part of the differential diagnosis of granuloma on MSGB [119,120]. Therefore, MSBG should only be considered in patients with elevated serum ACE or compatible CT abnormalities, where its performance is slightly better [118]. Endobronchial ultrasound-guided transbronchial lymph node aspiration has a good performance in the diagnosis of sarcoidosis in general [121]. However, very few data are available for ocular sarcoidosis [122].

### 5.5. An Algorithm for the Assessment of Patients with Suspected Sarcoid Uveitis

**Figure 5:**
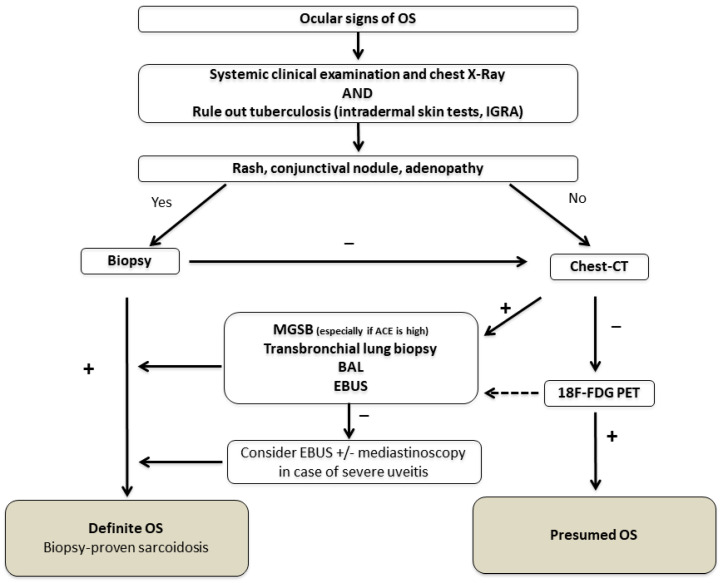
Diagnostic algorithm for suspected ocular sarcoidosis adapted from [74,123]. Abbreviations: ACE: angiotensin-converting enzyme; BAL: bronchoalveolar lavage; EBUS: endoscopic ultrasound-guided fine-needle aspiration of intrathoracic nodes; IGRA: interferon-γ release assay; MSGB: minor salivary-gland biopsy; OS: ocular sarcoidosis; 18F-FDG PET: 18-fluorodeoxyglucose positron emission tomography.

To sum up, our group has proposed a diagnostic strategy with simple or non-invasive biological and radiological investigations that can be followed by the more complex investigations required if there is posterior segment involvement worsening the visual prognosis or an indication for systemic treatment (Figure 5) [74].

## 6. Visual Prognosis of Sarcoid Uveitis

The visual prognosis for ocular sarcoid uveitis is generally good [124]. Fewer than 10% of patients have severe visual impairment, defined as visual acuity below 20/200 [37,38,125,126]. Complications of ocular inflammation are very frequent, in particular cataracts, which affect up to 73% of patients depending on the study [124,127]. In the report by Suzuki et al., cataract occurred in 62.2% of cases, glaucoma in 28.5%, epiretinal membrane in 24.1% and cystoid macular edema in 22.6% [124]. Nevertheless, the main cause of vision loss remains cystoid macular edema (as a consequence of uveitis) [37]. Several risk factors have been associated with poor functional prognosis such as a late-age onset, an African American origin, female sex, underlying chronic systemic sarcoidosis, posterior segment involvement, chronic cystoid macular edema, multifocal choroiditis, persistent ocular inflammation and glaucoma [12,28,38,41,124,128]. In a French tertiary center, slightly more than a quarter of the patients recover from their disease; two variables are associated with this favorable outcome: a Caucasian origin and an anterior location of the uveitis [40]. For women of childbearing age, the evolution during pregnancy seems reassuring even if few data exist. Our recent retrospective work suggests that it evolves in the same way as other uveitis (i.e., with a decreased frequency of relapses in the last trimester) [129].

## 7. Treatments

**Figure 6:**
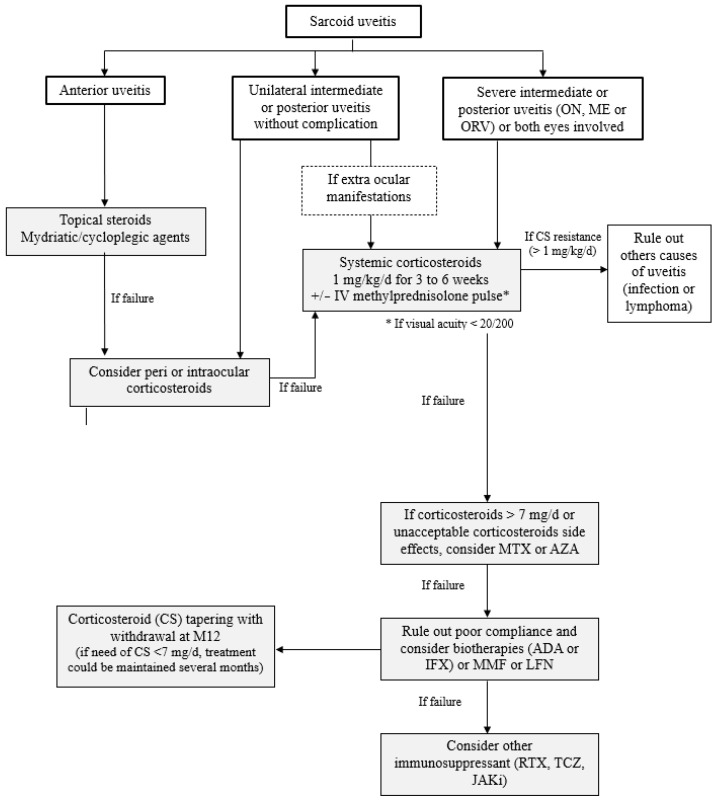
Therapeutic management in ocular sarcoidosis adapted from [123]. Abbreviations: ON: optic neuritis; ME: macular edema; ORV: occlusive retinal vasculitis; MTX: methotrexate; AZA: azathioprine; MMF: mycophenolate mofetil; LFN: leflunomide; RTX: rituximab; TCZ: tocilizumab; JAKi: Janus kinase inhibitor; IV: intravenous.

The level of evidence in the literature on the management of sarcoid uveitis is low and is mainly based on small retrospective studies [123]. The recent recommendations of the European Respiratory Society (ERS), based on the GRADE methodology and drafted with a committee of clinicians, methodologists and patients, did not take a position on the treatment of ocular sarcoidosis. Indeed, the expert group judged that the scientific data remained insufficient at the moment, with a particular absence of a study specific to ocular sarcoidosis [130]. In contrast, the therapeutic management of sarcoid uveitis was discussed during the 7th IWOS and recommendations according to anatomical type, based on the opinion of 13 experts, were published in 2020 [131]. The treatment of uveitis is medical, but surgery may be necessary to treat complications such as cataract or glaucoma [18,132]. In cases of epiretinal membrane with decreased visual acuity despite good control of ocular inflammation, vitrectomy with membranectomy may be beneficial [133]. Local or systemic corticosteroid therapy is the cornerstone of treatment for sarcoid uveitis. In refractory or cortico-dependent forms, immunosuppressive or biological treatment is sometimes used. In observational studies, almost all patients receive local treatment, whereas 45–70% require systemic treatment either because of ocular involvement or systemic disease [123]. We will detail the management according to the anatomical type of uveitis and discuss immunosuppressants and biological treatments (Figure 6).

### 7.1. Anterior Uveitis

Local treatment is the first-line treatment for anterior uveitis [131]. Topical ophthalmic solutions are often combined with mydriatic/cycloplegic agents to limit synechia. Second-line treatments include corticosteroid eye drops, dexamethasone subconjunctival injection, triamcinolone acetonide periocular injection and, as a last resort, systemic corticosteroids [131]. Systemic corticosteroid therapy is only appropriate as a second-line treatment for severe anterior uveitis [131].

### 7.2. Intermediate and Posterior Uveitis

The decision to treat intermediate and posterior uveitis depends not only on symptomatology but also on visual acuity and anatomical involvement such as macular edema, severity of retinal vasculitis or active choroiditis [131]. Topical corticosteroids are not efficient in treating the posterior segment [134,135]. According to the IWOS experts, intermediate uveitis (uni- or bilateral) can be treated locally (periocular, intravitreal, implant) or with systemic steroids [131]. The MUST trial, which compared systemic treatment in intermediate and posterior non-infectious uveitis with dexamethasone intravitreal implants, showed no difference in visual acuity at 24 months [136]. However, this study was not specific to sarcoidosis. Systemic treatment is preferred in cases of severe bilateral involvement, severe glaucoma and in young phakic patients. The first-line treatment of active posterior uveitis (macular edema, papillary nodules/granulomas, periphlebitis, peripheral chorioretinal lesions, choroidal nodules) includes systemic corticosteroid therapy alone or in combination with an immunosuppressive therapy and local corticosteroid therapy [131]. Argon laser photocoagulation is required for retinal ischaemia, whereas intravitreal injections of anti-vascular endothelial growth factor (VEGF) in combination with anti-inflammatory therapy is the treatment for choroidal neovascularization [18].

In practice, dexamethasone intravitreal implants (Ozurdex^®^, Abbvie, North Chicago, IL, USA) are currently taking over from subconjunctival injections with a duration of action of 4–6 months compared with 3 weeks [134,137,138]. Fluocinolone acetonide intravitreal implants (Iluvien^®^ Alimera Sciences Inc., Alpharetta, GA, USA) cover a period of 3 years and could decrease the frequency of non-infectious uveitis [139]. However, no specific data on sarcoidosis exist to our knowledge. The dosage of systemic corticosteroids ranges from 0.5–1 mg/kg up to a maximum of 80 mg/d, with an initial treatment of 2–4 weeks before starting a 3–6 month taper according to IWOS experts [131]. In the most severe forms of uveitis, the use of intravenous corticosteroid pulses can be discussed, but the level of evidence remains low [140,141].

### 7.3. Immunosuppressive Agents and Biologics

In 5–27% of cases, cortisone-sparing therapy is required, either because of high-dose corticosteroid dependence (more than 7.5–10 mg/day of prednisone equivalent needed to control the disease) or because of the side effects of systemic corticosteroid therapy [37,49,55]. The IWOS recommendations include the use of methotrexate, azathioprine, mycophenolate mofetil and ciclosporin. Biologics are then used as third-line therapies if necessary, particularly in the case of posterior uveitis [131]. The choice of the immunosuppressive agent must be made in agreement with the patient after discussion of their comorbidities and life plans (particularly the desire for pregnancy in young women). In general, methotrexate remains the cortisone-sparing treatment for which there is the most data on sarcoidosis in the literature [130]. In a single-center retrospective study of 50 patients with sarcoidosis, Baughman et al. showed a response in two-thirds of patients after six months of treatment [142]. The same author, from the largest ocular sarcoidosis series (465 patients), reported that methotrexate was both effective (77% of 365 treated patients were still on methotrexate at the end of follow-up whereas only 7% had discontinued treatment due to ineffectiveness) and well tolerated (3.8% discontinued for toxicity) [143]. In the same series, azathioprine had similar efficacy but was less well tolerated: of the 68 patients treated, 46 (67.7%) were still on treatment at the end of the follow-up whereas 13 (19.1%) had stopped it due to toxicity [143]. Studies on mycophenolate mofetil are very scarce, limited to a small series of seven patients by Bhat et al. and case reports [144,145]. Leclercq et al. compared the efficacy of several immunosuppressants for the treatment of sarcoid uveitis affecting the posterior segment in a two-center study including 67 patients. The comparison of first-line treatments showed superiority of MTX over MMF in terms of risk of relapse and adverse events [146]. Data on cyclosporine are anecdotal, even though it is used in Japan [147,148]. Leflunomide has also been reported as an alternative to methotrexate in cases of intolerance. Recently, our team reported the use of hydroxychloroquine (HCQ) for the treatment of sarcoid uveitis despite the potential ocular toxicity of antimalarials. In a retrospective series of 27 patients, HCQ (mean duration of treatment of 20 months) resulted in a significant reduction in systemic corticosteroid therapy and the number of relapses. However, HCQ was discontinued in 12 patients during follow-up, including 8 for ineffectiveness [149]. Its use could be particularly interesting in anterior and intermediate uveitis, but larger prospective studies will first need to confirm the results [149]. Altogether, initial resistance to corticosteroids and conventional immunosuppressants should rule out non-compliance, infectious granulomatosis or lymphoma before initiating treatment with biologics [150].

The reference biological treatments are anti-TNF [131]. Paradoxically, there are cases of drug-induced sarcoid uveitis with anti-TNF in patients with rheumatoid arthritis, ankylosing spondylitis and juvenile idiopathic arthritis [151,152]. Adalimumab is often the first choice because it is administered subcutaneously, allowing for outpatient management. In practice, infliximab is often reserved as a second-line treatment but may be used in cases of doubtful compliance. Three randomized trials have confirmed the efficacy of adalimumab in non-infectious uveitis but without subgroup analysis in sarcoidosis [153,154,155]. These studies have shown a significant reduction in the risk of treatment failure, defined as recurrence of ocular inflammation, in patients treated with adalimumab. Of note, adalimumab was not more effective in the subgroup of patients receiving concomitant immunosuppressive therapy [153]. After failure of a conventional immunosuppressant, infliximab appears to have similar efficacy and safety to adalimumab [156]. In contrast, in a retrospective study from the same group, infliximab appears to be more effective than adalimumab for the treatment of vision-threatening uveitis, particularly in Behçet’s disease [157]. Etanercept should probably not be used in uveitis due to a lower response rate than other anti-TNF agents [158]. Sarcoidosis uveitis is no exception to this rule, and the work of Baughman et al. found no efficacy of this drug [159]. Further studies will also be needed to position certolizumab pegol and golimumab in the therapeutic arsenal [160,161,162,163]

The use of other biotherapies is more exceptional in sarcoid uveitis [164]. Tocilizumab, an IL-6 receptor inhibitor, was shown to improve visual acuity and reduced foveal thickness in non-anterior uveitis in the randomized open-label study STOP [165]. This drug shows interesting efficacy on macular edema [166]. The BIOVASC retrospective study, which included 29 cases of sarcoidosis among the 149 uveitis cases included, recently demonstrated superior efficacy of TCZ compared with anti-TNF-α in the treatment of refractory macular edema in terms of inflammatory response, with no difference in relapse and cortisone sparing [167]. Several authors have shown the efficacy of the Janus kinase (JAK) inhibitors tofacitinib or ruxolitinib in pulmonary and cutaneous sarcoidosis refractory to other drugs [168]. We report an observation of paradoxical sarcoidosis panuveitis induced by adalimumab in the context of rheumatoid arthritis. Although the uveitis and inflammatory rheumatism remained active upon discontinuation of anti-TNFα, the ocular inflammation and mediastino-hilar adenopathy disappeared with tofacitinib treatment [169]. Tofacitinib is currently being tested in non-infectious uveitis (NCT03580343).

## 8. Conclusions

Sarcoidosis is one of the most common causes of uveitis. Uveitis is the most common ophthalmological involvement in sarcoidosis, hence the requirement for knowledge on ophthalmological semiology in the management of sarcoidosis. The ophthalmic symptomatology should be known not only by ophthalmologists but also by all specialists dealing with patients with sarcoidosis. A close collaboration between the different specialists is necessary for both diagnosis and therapeutic management. Larger prospective studies are still needed to improve the level of evidence for the recommendations on the management of sarcoid uveitis.

## Figures and Tables

**Table 1:** Classification criteria for sarcoid uveitis as recommended by the “Standardization of Uveitis Nomenclature (SUN)”, reprinted from Standardization of Uveitis Nomenclature (SUN) Working Group Classification Criteria for Sarcoidosis-Associated Uveitis. *Am J Ophthalmol* **2021** [50], with permission from Elsevier.

Criteria
1. Compatible uveitic picture, either: a. Anterior uveitis or b. Intermediate or anterior/intermediate uveitis or c. Posterior uveitis with either choroiditis (paucifocal choroidal nodule(s) or multifocal choroiditis) or d. Panuveitis with choroiditis or retinal vascular sheathing or retinal vascular occlusion.
AND
2. Evidence of sarcoidosis, either: a. Tissue biopsy demonstrating non-caseating granulomata or b. Bilateral hilar adenopathy on chest imaging.
**Exclusions**
1. Positive serology for syphilis using a treponemal test. 2. Evidence of infection with *Mycobacterium tuberculosis* ^a^, either: a. Histologically or microbiologically confirmed infection with *M. tuberculosis* ^b^ or b. Positive interferon-γ release assay (IGRA) ^c^ or c. Positive tuberculin skin test ^d^

^a^ Routine exclusion of tuberculosis is not required in areas where tuberculosis is non-endemic but should be performed in areas where tuberculosis is endemic or in tuberculosis-exposed patients. With evidence of latent tuberculosis in a patient with a uveitic syndrome compatible with either sarcoidosis or tubercular uveitis and bilateral hilar adenopathy, the classification as sarcoid uveitis can be made only with biopsy confirmation of sarcoidosis (and therefore exclusion of tuberculosis). ^b^ For example, biopsy, fluorochrome stain, culture or polymerase-chain-reaction-based assay. ^c^ For example, Quantiferon-gold or T-spot. ^d^ For example, a purified-protein-derivative skin test positive result should be >10 mm induration.

**Table 2:** Diagnostic performances of the main biomarkers available in ocular sarcoidosis.

Biomarker	Test Performance	Comments	References
Lymphopenia *	Se: 0.75	Increased Sp (0.97) with 1000/mm^3^ cut-off. Increased Sp (0.99) and PPV (0.73) with elevated ACE. Easily accessible and non-invasive.	[88,93,104]
	Sp: 0.77		
	PPV: 0.48		
	NPV: 0.85		
Elevated ACE * (>52–61 UI/I)	Se: 0.38–0.84	Highly specific. Interesting to rule out OS diagnosis NPV (0.97). Uninterpretable if patient uses ACE inhibitors.	[84,90,91,93]
	Sp: 0.83–0.98		
	PPV: 0.44		
	NPV: 0.89–0.97		
Lysozyme *	Se: 0.6–0.78	High lysozyme levels can be found in infectious uveitis (tuberculosis, latent syphilis).	[84,94]
	Sp: 0.76–0.95		
sIL-2R (Threshold according to manufacturer)	Se: 0.69–0.94	The best test performance. No validated threshold. Not validated in revised IWOS criteria.	[95,96,97,98]
	Sp: 0.64–0.98		
	PPV: 0.77		
	NPV: 0.99		
Chitotriosidase activity	No data in sarcoidosis uveitis.	Elevated in systemic sarcoidosis and pulmonary diseases (COPD, asbestosis).	[102]

Abbreviations: Se: sensitivity; Sp: specificity; PPV: positive predictive value; NPV: negative predictive value; sIL-2R: soluble interleukin-2 receptor; ACE: angiotensin-converting enzyme; OS: ocular sarcoidosis; COPD: chronic obstructive pulmonary disease. * Biomarker included in the IWOS criteria.

## Data Availability

Not applicable.
